# Targeting Alpha-Synuclein as a Therapy for Parkinson’s Disease

**DOI:** 10.3389/fnmol.2019.00299

**Published:** 2019-12-05

**Authors:** Carroll Rutherford Fields, Nora Bengoa-Vergniory, Richard Wade-Martins

**Affiliations:** ^1^Department of Neuroscience at Johns Hopkins University, Baltimore, MD, United States; ^2^Department of Physiology, Oxford Parkinson’s Disease Center, Anatomy and Genetics, Oxford, United Kingdom

**Keywords:** alpha-synuclein, Parkinson’s disease, aggregation, therapy, oligomers, fibrils

## Abstract

Parkinson’s disease (PD) is one of the most common neurodegenerative disorders with a global burden of approximately 6.1 million patients. Alpha-synuclein has been linked to both the sporadic and familial forms of the disease. Moreover, alpha-synuclein is present in Lewy-bodies, the neuropathological hallmark of PD, and the protein and its aggregation have been widely linked to neurotoxic pathways that ultimately lead to neurodegeneration. Such pathways include autophagy/lysosomal dysregulation, synaptic dysfunction, mitochondrial disruption, and endoplasmic reticulum (ER) and oxidative stress. Alpha-synuclein has not only been shown to alter cellular pathways but also to spread between cells, causing aggregation in host cells. Therapeutic approaches will need to address several, if not all, of these angles of alpha-synuclein toxicity. Here we review the current advances in therapeutic efforts for PD that aim to produce a disease-modifying therapy by targeting the spread, production, aggregation, and degradation of alpha-synuclein. These include: receptor blocking strategies whereby putative alpha-synuclein receptors could be blocked inhibiting alpha-synuclein spread, an alpha-synuclein reduction which will decrease the amount alpha-synuclein available for aggregation and pathway disruption, the use of small molecules in order to target alpha-synuclein aggregation, immunotherapy and the increase of alpha-synuclein degradation by increasing autophagy/lysosomal flux. The research discussed here may lead to a disease-modifying therapy that tackles disease onset and progression in the future.

## Introduction

Parkinson’s disease (PD) is the second most common neurodegenerative disorder, after Alzheimer’s Disease, and the most common movement disorder (Mhyre et al., 2012). PD has a prevalence of approximately 0.5–1% among individuals 65–69 years of age, rising to 1–3% among persons 80 years of age and older (Nussbaum and Ellis, 2003). PD is characterized by motor symptoms including tremor, rigidity, bradykinesia, postural instability, gait and balance impairment and non-motor symptoms such as cognitive decline, autonomic impairment, rapid eye movement behavioral sleep disorder, constipation, and other behavioral disturbances (Stacy, 2011; Obeso et al., 2017). Additionally, its hallmark feature is the accumulation of alpha-synuclein (α-syn) that results in the formation of proteinaceous cytoplasmic inclusions, known as Lewy-bodies and Lewy neurites (LBs/LNs; Jakes et al., 1994). Current therapeutic approaches are directed at controlling symptoms and delaying the progression of the disease for as long as possible (Poewe, 2009). Therefore, the development of disease-modifying therapeutics is extremely attractive in experimental and clinical research in PD.

Multiple lines of evidence support the critical importance of α-syn in PD pathogenesis. The intraneuronal proteinaceous cytoplasmic inclusions now known as LBs, the hallmark of PD, were first described by Lewy in 1912 (Lewy, 1912; Goedert et al., 2013). Several decades later, in 1996, the first link between α-syn and the PD phenotype was established with the identification of the A53T point mutation on chromosome 4q21-23 (Polymeropoulos et al., 1996). The gene encoding α-syn (*SNCA*) was identified the following year (Polymeropoulos et al., 1997). Subsequently, α-syn was identified as a major component of LBs and LNs (Spillantini et al., 1997). While we have since achieved a greater understanding of PD pathogenesis, the exact mechanisms elucidating the nature of progressive dopaminergic cell loss in the substantia nigra (SN) pars compacta remain to be determined. In this review article, we examine advances in our current understanding of the pivotal role of α-syn in PD pathogenesis, including pathways implicated in α-syn toxicity, suggested seeding and propagation mechanisms that underlie cell-to-cell transmission between neighboring neurons, and viability of potential disease-modifying therapeutics targeted against pathological α-syn species.

### α-Syn

Although the full extent of the physiological function for α-syn is yet to be revealed and there may be conflicting findings in need of resolution, α-syn is involved in synaptic activity through regulation of vesicle docking, fusion, and neurotransmitter release (Ghiglieri et al., 2018). α-syn is an abundant 14 kDa protein consisting of 140 amino acids and comprised of three domains: (1) an N-terminal lipid-binding alpha-helix; (2) a non-amyloid-component (NAC); and (3) an acidic C-terminal tail (Lashuel et al., 2013). The N-terminal domain of α-syn is characterized by a series of seven 11-residue imperfect repeats, each based upon a highly conserved KTKEGV hexameric motif that is also observed in the α-helical domain of apolipoproteins (Davidson et al., 1998; Bussell and Eliezer, 2003; Bussell et al., 2005). This similar architecture allows α-syn to mediate lipid interactions in a similar way to how apolipoproteins do, inserting its amphipathic helices into lipid membranes to influence their curvature (Davidson et al., 1998). The central region (residues 61–95), also known as the NAC domain, can form cross β-sheets and consists of a highly hydrophobic sequence underlying its high propensity for aggregation and leading to protofibril and fibril formation (Uéda et al., 1993; Giasson et al., 2001; Tuttle et al., 2016). The predominantly unstructured conformation of α-syn makes it a target for various post-translational modifications such as phosphorylation. Indeed phosphorylation of Serine 129 in the C-terminal domain of α-syn has been associated with an increased propensity of aggregate formation (Samuel et al., 2016). A number of studies have reported aberrant accumulation of phosphorylated α-syn at the Serine-129 residue (pS129) in the brains of PD patients, as well as in animal models of synucleinopathies (Tenreiro et al., 2014; Oueslati, 2016). While only a small fraction of α-syn (~4%) is phosphorylated in healthy brains, substantial accumulation of pS129 (~90%) is observed in brains with Lewy pathology, implicating a potentially important association between this posttranslational modification and the accumulation of α-syn aggregates concomitant with LB formation and neurodegeneration (Fujiwara et al., 2002; Hasegawa et al., 2002). Further insight on the significance of S129 phosphorylation on α-syn aggregation, LB formation, and neurotoxicity may provide insight into the nature of PD pathogenesis and PD and related disorders. While numerous lines of evidence indicate that monomeric α-syn is not toxic, several studies highlight α-syn oligomers and fibrils as the species responsible for α-syn toxicity (Neumann et al., 2004; Outeiro et al., 2008; Peelaerts et al., 2015), and it has been shown that overexpression or triplication of synuclein is sufficient for α-syn aggregation to take place (Outeiro et al., 2008; Zambon et al., 2019).

There are conflicting findings on the nature of the native state of α-syn. Although the majority of studies suggest that cytosolic α-syn is present within cells as an intrinsically unfolded monomer, alternative hypotheses have proposed that it exists as a tetrameric alpha-helical oligomer that is resistant to fibrillization and thus distinct from pathological variants (Bartels et al., 2011; Wang et al., 2011; Fauvet et al., 2012; Burré et al., 2013; Smaldone et al., 2015). α-Syn can adopt an α-helical conformation in association with biological membranes or remain in an intrinsically unfolded state in the cytosol, suggesting it has different functions in different subcellular locations depending on its dynamic structure (Eliezer et al., 2001; Ramakrishnan et al., 2006; Ullman et al., 2011). Indeed, a recent investigation lends further support to this notion using chemical crosslinking and FRET experiments to demonstrate that α-syn multimerizes into a tetrameric complex upon binding cellular membranes and that it is this tetrameric membrane-bound α-syn that mediates SNAP receptor (SNARE) complex assembly and that functions as a molecular chaperone for these complexes at the presynaptic membrane (Burré et al., 2014). These findings should be interpreted with caution as evidence for tetrameric α-syn stems from crosslinked samples.

## Pathways Implicated in Toxicity of α-Syn

**Figure 1:**
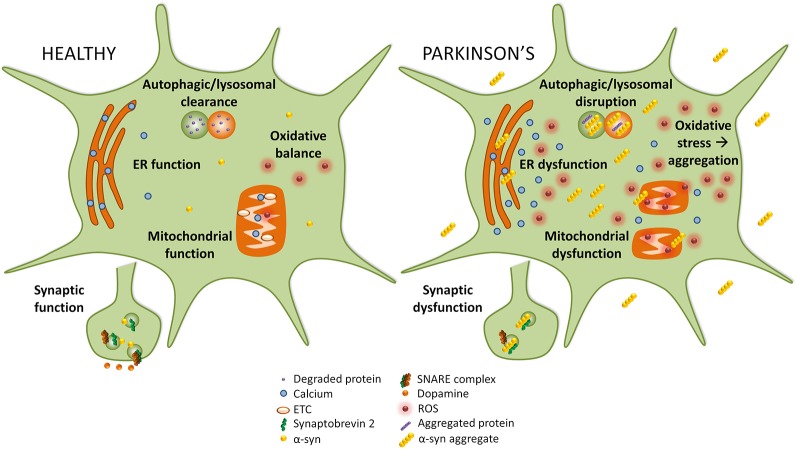
Implicated pathways for α-syn toxicity. To the left, healthy cellular pathways are illustrated, while to the right examples of how these pathways are perturbed in Parkinson’s disease (PD) are shown. Under normal circumstances, autophagic and lysosomal clearance degrades protein and other debris in the cell. In PD these pathways are blocked causing an accumulation of aggregated protein that could itself lead to more aggregation. In healthy cells, endoplasmic reticulum (ER) function is preserved, but in PD ER stress leads to calcium efflux into the cytoplasm. While in healthy cells mitochondrial function and oxidative balance are maintained, in PD the electron transport chain (ETC) and mitochondria function are compromised which causes an increase in reactive oxygen species (ROS) that leads to oxidative stress. Finally, in PD α-syn may interact with synaptobrevin-2, leading to synaptic dysfunction.

Disruption of several cellular pathways leads to the loss of dopaminergic neurons in PD, including synaptic vesicle recycling, mitochondrial function, oxidative stress, endoplasmic reticulum (ER) stress, and autophagy-lysosomal pathway (ALP) function (Figure 1). The accumulation of α-syn into prefibrillar forms, and then its assembly into higher molecular weight aggregates, induces cellular toxicity and maybe the greatest contributor to pathogenesis in PD (Bengoa-Vergniory et al., 2017). The increased cellular toxic burden caused by aggregated α-syn may arise from overexpression of the protein, genetic multiplication, or impairment to normal protein clearance mechanisms such as autophagy (Alegre-Abarrategui et al., 2019).

### Synaptic Vesicle Impairment

α-syn is typically localized to the presynaptic terminal where it associates with synaptic vesicles and influences membrane curvature (Maroteaux et al., 1988; Wong and Krainc, 2017). There it is known to promote synaptic vesicle fusion and other processes in synaptic-vesicle trafficking through its interactions with the synaptobrevin-2 component of the SNARE (soluble N-ethylmaleimide-sensitive factor attachment protein receptors) complex. Large α-syn oligomers preferentially bind synaptobrevin-2 and may disrupt SNARE complex assembly, synaptic-vesicle motility, and dopamine release (Burré et al., 2010; Choi et al., 2013). Additionally, loss of α-syn has been associated with an increase in dopaminergic release (Senior et al., 2008; Anwar et al., 2011), and so the aggregation of α-syn could also lead to loss of function effects at the synapse.

### Mitochondrial Dysfunction

Dopaminergic neurons are uniquely susceptible to mitochondrial dysfunction due to their high energy demands and increased exposure to oxidative stress (Valente et al., 2004; Ricciardi et al., 2014). The selective vulnerability of these neurons was first recognized following the finding that the mitochondrial neurotoxin 1-methyl-4-phenyl-1,2,3,6-tetrahydropyridine (MPTP) resulted in the cell death of SN DA neurons in humans (Langston et al., 1983). Moreover, MPTP was shown to be toxic to DA neurons in mouse and non-human primate models of PD (Przedborski et al., 2001). α-Syn oligomers can also inhibit the import of proteins, including some subunits of complex I, into the mitochondria by binding to the translocase of the outer membrane (TOM20) and inhibiting its interaction with the co-receptor TOM22 (Di Maio et al., 2016). Overexpression of the α-syn mutants A53T or A30P has been shown to increase the aggregation of α-syn in human neuroblastoma cells (Parihar et al., 2009). In these cells, immunogold electron transmission microscopy revealed the localization of α-syn aggregates within the mitochondria of overexpressing cells, which exhibited decreased mitochondrial transmembrane potential and limited cellular respiration concomitant with increased production of reactive oxygen species (ROS). The proximity between the mitochondrial electron transport chain (ETC) and mitochondria DNA (mtDNA) increases the vulnerability of mutations in mtDNA due to ROS, especially during mitochondrial dysfunction, such as in complex I inhibition (Davis et al., 1979). Indeed, mutations in mtDNA or impairment to the ETC cause mitochondrial dysfunction and energy depletion. Damaged mitochondria result in electron leakage, produce increased ROS, and release cytochrome C leading to activation of caspase-3, caspase-9, and other pro-apoptotic factors ultimately leading to cell death (Brustovetsky et al., 2002). Additionally, α-syn can interfere with mitochondrial membrane fusion and fission, resulting in fragmentation of mitochondria, and it can inhibit mitophagy and complex I activity, and disrupt mitochondrial membrane potential (Cole et al., 2008; Devi et al., 2008; Chen and Chan, 2009). While this review concentrates on PD it is worth noting that oligodendrocytes, which are heavily loaded with pathological glial cytoplasmic inclusions in multiple system atrophy (MSA) patients, are also cells with high endogenous α-syn and a high metabolic profile. It is interesting to consider the parallels between these two synucleinopathies and cellular selective vulnerability (Alegre-Abarrategui et al., 2019).

### Oxidative Stress

Failure of the antioxidant proteins regulating ROS levels, like superoxide dismutase (SOD) and glutathione (GSH), results in oxidative stress, which may have deleterious effects inside cells (Indo et al., 2015). Interestingly, the SN appeared to contain twice as much oxidized proteins as compared with the caudate, putamen, and frontal cortex in the post-mortem brains of healthy individuals, suggesting that the increased susceptibility of SN to oxidative stress may contribute to the selective degeneration of nigral DA neurons (Floor and Wetzel, 2002). Unregulated oxidation of intracellular macromolecules and organelles can cause cellular damage and may lead to cell death (Wiseman and Halliwell, 1996; Rego and Oliveira, 2003). As one of the major producers of ROS, mitochondria are particularly vulnerable to oxidative stress-induced cytotoxicity, especially considering mtDNA is not protected by histone proteins as seen in nuclear DNA (Richter et al., 1988). As mentioned above, α-syn can interfere with translocation of mitochondrial-target proteins, thereby disrupting the proper functioning of the ETC, and leading to elevated levels of intracellular oxidative stress (Di Maio et al., 2016). Interestingly, while α-syn toxicity is implicated in increasing cellular oxidative stress, it has also been suggested that oxidative stress can induce α-syn toxicity. Excessive exposure to oxidative stress causes lipid peroxidation of polyunsaturated fatty acids, which in turn leads to the formation of 4-hyroxy-2-nonenal, a product that has been shown to hamper fibrillization of α-syn and promote the formation of secondary beta sheets and toxic soluble oligomers in a dose-dependent manner (Dexter et al., 1989; Qin et al., 2007; Bae et al., 2013). Incubation of α-syn with cytochrome c/H_2_O_2_ leads to the oxidative stress-induced aggregation of α-syn by crosslinking α-syn tyrosine residues through dityrosine bonding (Hashimoto et al., 1999; Ruf et al., 2008). Moreover, colocalization of cytochrome c and α-syn was reported in the LB of patients with PD (Hashimoto et al., 1999).

### Endoplasmic Reticulum (ER) Stress

The ER is essential for the synthesis, modification, and delivery of proteins to their target sites within the secretory pathway (i.e., Golgi). Overexpressed or mutant α-syn accumulates within the ER, interfering with protein folding and inducing ER stress, which may contribute to neurodegeneration (Colla et al., 2012). Smith et al. reported that increased ROS levels stemming from ER stress and mitochondrial dysfunction contribute to A53T α-syn-induced cell death (Smith et al., 2005). Moreover, aggregated α-syn impairs both the ubiquitin-proteasome system and autophagy, resulting in ER stress and the activation of the unfolded protein response (UPR; Bence et al., 2001; Xu et al., 2005; Kim et al., 2008). Accumulation of α-syn in the ER results in calcium leakage into the cytosol, which then acts on α-syn in a feedback-like manner potentiating further aggregation (Volles and Lansbury, 2002; Kayed et al., 2004; Sokolov et al., 2006; Nath et al., 2011). Mitochondria also function in regulating calcium homeostasis. Increased mitochondrial uptake of cytosolic calcium released by the ER generates excessive ROS (Nunnari and Suomalainen, 2012; Görlach et al., 2015; Paupe and Prudent, 2018). Additionally, the protein folding capacity of the ER operates in an ATP-dependent manner, thus sustained UPR activation promotes increased ROS production.

### Autophagy-Lysosomal Pathway (ALP) Dysfunction

α-Syn overexpression also disrupts autophagy, a cellular process involved in the degradation of damaged organelles, invading microorganisms and aggregated proteins (Wong and Holzbaur, 2015). Impairment of autophagic processes is reported to result in the accumulation of α-syn and propagation in a prion-like fashion, further potentiating the cellular toxicity of α-syn pathology. In addition, A53T and A30P α-syn exhibit a stronger binding affinity for the lysosomal receptor LAMP2A compared with wild-type (WT) α-syn, so these mutant forms of α-syn are not efficiently degraded by protein clearance mechanisms, which results in increased α-syn burden of chaperone-mediated autophagy and inhibits the loading and clearance of other cargo (Cuervo et al., 2004). Moreover, α-syn overexpression in iPSCs compared with controls reduced the enzymatic activity of multiple lysosomal enzymes, including GCase, which is essential for the proper functioning of the autophagolysosome (Mazzulli et al., 2011, 2016). Mutations in the leucine-rich repeat kinase 2 (LRRK2) protein may also disrupt autophagy and lysosomal function and are the most common cause of familial and sporadic PD. Elevated mutant LRRK2 kinase activity is associated with cytotoxicity (West, 2017). Jeong et al. (2018) demonstrated that dysregulation of downstream Rab substrates of LRRK2 resulted in neurodegeneration of dopaminergic neurons in the mammalian brain. Their findings suggest further study of the Rab GTPases may not only elucidate the processes governing these intercellular membrane dynamics, but also reveal molecules or pathways that can serve as potential targets for therapeutic intervention. Indeed, a recent study showed that the accumulation of phospho-α-syn in a rat rotenone model correlated with ALP dysfunction and that LRRK2 inhibitors could prevent these effects (Di Maio et al., 2018).

## Cell-to-Cell Transmission of α-Syn

α-Syn is proposed to propagate from the peripheral (i.e., enteric) nervous system (PNS) to the central nervous system (CNS) as well as spread *via* a cell-to-cell transmission (Volpicelli-Daley and Brundin, 2018). In 2003, a seminal study published by Braak et al. (2003) introduced a six-stage system for PD based on the observed caudo-rostral pattern of progression of α-syn pathology, with stage 1 originating in the lower brainstem and stage 6 extending to involve the cortex. According to this theoretical caudo-rostral pattern of progression, the olfactory system, caudal brainstem, and autonomic nervous system were among the earliest areas affected by α-syn pathology (Braak and Braak stages 1 and 2). This was followed by a significant loss of dopaminergic neurons in the SN (Braak and Braak stages 3 and 4), and subsequent extensive cortical involvement (Braak and Braak stages 5 and 6). Consistent with this hypothesis were the findings that patients who have undergone vagotomy (Svensson et al., 2015) or appendectomy (Killinger et al., 2018) have reduced risk of developing PD. However, not all cases of sporadic PD exhibit α-syn pathology as predicted based on the suggested anatomical hierarchy of the caudo-rostral progression pattern of pathology (Burke et al., 2008; Alafuzoff et al., 2009). Additionally, Braak and Braak’s staging system does not adequately explain the absence of clinical symptoms in individuals with observable widespread α-syn pathology at autopsy (Parkkinen et al., 2005; Alafuzoff et al., 2009). A retrospective autopsy series in 30–55% of elderly subjects with widespread Lewy-related pathology (Braak and Braak stages 5 and 6) reported no definite neuropsychiatric symptoms, suggesting considerable cerebral compensatory mechanisms (Jellinger, 2008). Nevertheless, Braak and Braak’s model has successfully demonstrated that the α-syn pathology present in PD is not only restricted to the SN but extends to involve several other brain regions and both the PNS and CNS. Although the precise mechanisms underlying disease progression are yet to be established, pathology could originate in the gut and proceed retrogradely to the brain *via* the vagal nerve or could start in the vagal nerve and extend to the gut *via* anterograde movement (Braak and Del Tredici, 2016; Kim et al., 2019).

Further evidence supporting the hypothesis that α-syn may self-propagate and spread progressively between interconnected brain regions through a cell-to-cell transmission mechanism came from the pathological analysis of grafted nigral neurons. In 2008, two independent postmortem studies reported that healthy embryonic mesencephalic neurons grafted into the striatum of PD patients developed α-syn pathology or LB-like structures many years after brain surgery (Kordower et al., 2008; Li et al., 2008). These findings suggested host-to-graft propagation of α-syn pathology and gave rise to the idea of a “prion-like” transmission mechanism to describe the pathogenic potential of disease progression. In this model, neuron-released aggregated α-syn in the extracellular space may be internalized by neighboring neurons, where it may act as a seed to induce further misfolding and aggregation of endogenous α-syn proteins. Repeated subsequent cycles of α-syn aggregate formation and release are thought to correspond with further disease progression (Brettschneider et al., 2015).

Multiple pre-clinical studies both *in vitro* and *in vivo* have demonstrated strong evidence supporting prion-like propagation and transmission of α-syn (Spillantini et al., 1998; Prusiner et al., 2015). Desplats et al. (2009) were one of the first studies to demonstrate a cell-to-cell transmission mechanism of α-syn *in vivo*. The study reported human α-syn transfer in 15% of the fluorescently labeled mouse neural stem cells transplanted into the hippocampus of α-syn transgenic mice. In a recent study, injection of nigral LB-enriched fractions containing pathological α-syn was purified from postmortem PD brains and inoculated into the SN or striatum of WT mice and rhesus macaque monkeys. In both mice and monkeys, intranigral or intrastriatal inoculation of PD-derived LB extracts resulted in progressive nigrostriatal neurodegeneration starting at striatal dopaminergic terminals. At the onset of LB-induced degeneration, host pathological α-syn diffusely accumulated within nigral neurons and anatomically linked brain regions (Recasens et al., 2014). Subsequent *in vivo* studies using an injection of recombinant α-syn aggregates further support the hypothesis of cell-to-cell transmissibility of pathogenic α-syn. Through injection of α-syn preformed fibrils (PFFs) into the striatum of transgenic mice, researchers demonstrated the development of Lewy pathology, nigrostriatal degeneration, and importantly expanded our understanding of cell-to-cell transmission by describing the nature of spread in neuroanatomically connected regions: this provided the first evidence that synthetic α-syn PFFs alone can induce the initiation and propagation of α-syn pathology *in vivo* (Luk et al., 2012). Furthermore, intracerebral injections of recombinant human or mouse fibrils directly into the SN of C57BL/6J mice or into asymptomatic transgenic mice induced a time-dependent development of extensive α-syn pathology (Masuda-Suzukake et al., 2013). Similar observations are reported in rats after nigral inoculation with four different structural types of α-syn assemblies: two distinct strains denoted “fibrils” and “ribbons,” α-syn oligomers, and brain homogenates from transgenic mice expressing mutant human α-syn (A30P; Peelaerts et al., 2015). Interestingly, these findings suggest there might be distinct properties to the different strains of α-syn aggregates associated with PD pathology, including seeding propensity, rate of aggregation, and potential to trigger an inflammatory response.

The evidence of cell-to-cell transmission of pathologic α-syn in interconnected brain regions suggests a prion-like mechanism of spread. However, a recent study demonstrated that the spread of α-syn pathology does not always proceed as expected along the connectome, either anterogradely or retrogradely, through a template-recruitment process reminiscent of that observed in prion diseases (Sorrentino et al., 2017). Furthermore, although injected fibrillar human α-syn induced extensive α-syn pathology and protein inclusions in A53T transgenic mice, in E46K transgenic mice α-syn pathology was predominantly localized to the site of injection with no evidence for spread (Sacino et al., 2014). Importantly, it is worth noting that the Mendez et al.’s (2008) study reported no evidence of LB pathology in the surviving grafts 14 years after graft transplantation. Although there is significant evidence supporting the concept of cell-to-cell propagation of α-syn pathology, there are some discrepancies that need to be taken into consideration, such as differences in the experimental paradigm, starting material for the injection, the injection/graft environment, observational period post-injection, animal models used, and individual differences between PD patients.

## Therapeutic Approaches Targeting α-Syn for PD Treatment

**Figure 2:**
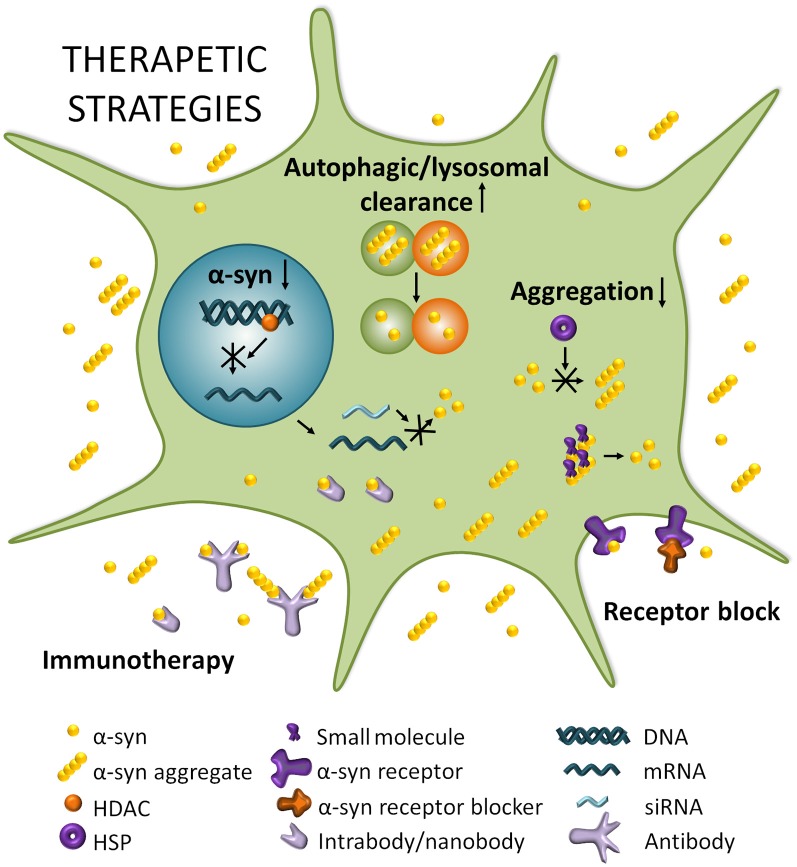
Therapeutic strategies for PD. Boosting autophagic/lysosomal clearance is a potential avenue for clearing α-syn and other aggregating proteins that disrupt cellular homeostasis. Reducing *SNCA* mRNA by modulating histone deacetylase (HDACs) or through RNA interference (RNAi) strategies can potentially lead to a decrease in expression of α-syn which is known to result in reduced aggregation and toxicity. Reducing aggregation can be achieved by impeding the multimerization of α-syn through heat shock proteins (HSPs) for example, or by dissociating existing aggregates with small molecules. Blocking α-syn entry through receptor blocking would directly target the spread of α-syn and prevent its transport from cell to cell. Finally, immunotherapy could potentially neutralize α-syn and/or α-syn aggregates extracellularly and perhaps even intracellularly in the case of intra/nanobodies.

There is currently no disease-modifying therapies for PD, but medication or surgery can provide palliative treatment directed at controlling symptoms that may substantially improve motor impairments. A systematic division of different strategies to target α-syn thus separates stabilizing the physiological conformation of α-syn, decreasing its expression, inhibiting its aggregation, and increasing intracellular clearance, from transmission-directed approaches including inhibiting uptake by neighboring cells and enhancing extracellular clearance mechanisms (Figure 2; Kaufman and Diamond, 2013; Hasegawa et al., 2017).

### Interference With the Prion-Like Spread of α-Syn

Ongoing research seeks to identify disease-modifying agents for PD that will restrict templated conformation changes and transcellular propagation of pathological α-syn. However, as endogenous α-syn is necessary in order for α-syn to spread (Braak et al., 2003; Kordower et al., 2008; Li et al., 2008), it is difficult to only specifically target the spread of α-syn, and so multiple approaches rely on targeting α-syn that is associated with its spread, while also partially targeting endogenous α-syn. An efficient method to regulate transmission would be blocked α-syn receptors; blocking the LAG3 cell-surface protein, an immune receptor involved in the endocytosis of extracellular aggregated α-syn (Mao et al., 2016) would be an interesting therapeutic avenue. LAG3-directed antibodies significantly reduce misfolded α-syn-induced toxicity and transmission (Anderson et al., 2016; Mao et al., 2016). However, LAG3 expression was not associated with modified disease progression in a different study (Liu et al., 2018). One influential study proposed that heparan sulfate proteoglycans on the surfaces of cells can mediate the uptake of amyloid fibrils, including those composed of α-syn, through endocytosis (Holmes et al., 2013). Thus, limiting the endocytosis of extracellular α-syn may hamper its pathogenic seeding potential and delay the progression of Lewy pathology. Although it remains unclear how α-syn escapes the endosome, it has been suggested that compromised endo-lysosomal membrane integrity increases cell susceptibility to α-syn aggregation after internalization of seeds *via* endocytosis (Jiang et al., 2017). Cell cultures treated with heparin and chloral hydrate, which both disrupt heparan sulfate proteoglycans, suffered a reduction of endocytic uptake of α-syn (Holmes et al., 2013). Further experiments should be conducted in animal models to identify specific inhibitors of heparan sulfate proteoglycans that can slow the pathology propagation cycle without interfering with essential cellular processes.

A careful study of these and other potential α-syn receptors is warranted in order to advance α-syn receptor-blocking therapies.

### Reducing α-Syn Production

Since α-syn gene duplications and triplications lead to PD, a potential therapeutic approach is to reduce α-syn production. This reduction of total protein amounts can be achieved with RNA interference (RNAi), using gene-silencing mechanisms to target α-syn mRNA levels. Indeed, silencing α-syn has led to neuroprotection *in vitro* upon 1-methyl-4-phenylpyridinium (MPP+) insult (Fountaine and Wade-Martins, 2007). Also, shRNA delivered *via* a lentiviral vector silenced human α-syn expression in rat striatum, and siRNA directed against α-syn reduced the expression of endogenous α-syn after 2 weeks of infusion into the mouse hippocampus (Sapru et al., 2006; Lewis et al., 2008). The success of these studies prompted testing of chronic siRNA infusions directed against α-syn in squirrel monkeys (McCormack et al., 2010). Following a unilateral infusion, α-syn levels were reduced by 40–50% relative to the untreated side. Additionally, experiments in rodent models demonstrated that antisense oligonucleotides (ASOs) safely reduced levels of α-syn protein expressions and did not affect the normal nigral dopaminergic neuronal function or cause neurodegeneration (Alarcón-Arís et al., 2018). However, a major challenge is that the precise physiological role of α-syn has not yet been characterized, and thus a therapy aimed at a reduction of its expression could have a significant impact. Indeed, Manfredsson and colleagues reported that the marked reduction (>90%) of α-syn achieved using viral vectors in rat and non-human primate SN corresponded to nigrostriatal system degeneration (Gorbatyuk et al., 2010; Kanaan and Manfredsson, 2012; Collier et al., 2016). It is essential to determine the degree of reduction necessary to effectively prevent α-syn accumulation in preclinical trials before this approach is considered viable for further investigation in PD patients. Additionally, careful consideration must be taken regarding the method of delivery, as systemic administration of RNAi or ASOs could potentially act on α-syn expressed in peripheral tissues. Hence, it is essential to perform further experiments in preclinical trials, especially in non-human primates, to clearly determine preliminary efficacy, tolerability and safety profile of this therapeutic approach, before preparations can be made to move into the clinical stage.

In addition to targeting α-syn mRNA translation processes, an alternative approach to reduce α-syn protein expression seeks to interfere with the transcription of the α-syn gene. A recent study found that β2-adrenoreceptor (B2AR) agonists (e.g.,: clenbuterol and salbutamol) could reduce α-syn gene expression by modulating transcription through altering histone deacetylase (HDAC) activity at the α-syn gene promoter and enhancer regions, and found that these modifications were neuroprotective in cell line and rodent models (Mittal et al., 2017). Consistent with these findings, a Norwegian epidemiological study reported that treatment with the B2AR salbutamol against asthma corresponded with a reduced lifetime risk of developing PD, whereas the B2AR antagonist propranolol purportedly increased PD risk (Mittal et al., 2017). This report indicates that B2AR agonists may show promise as potential disease-modifying agents that may inhibit α-syn protein expression at the transcriptional level. Further studies should be pursued in animal models of α-syncleinopathies to investigate therapeutic implications for PD patients.

### Inhibition of α-Syn Aggregation and Aggregate Reduction

Inhibition of α-syn aggregation remains an extremely attractive potential target for therapeutic interventions. Heat shock proteins (HSP) act as molecular chaperones that assist nascent polypeptide chains to fold correctly, and thus help prevent protein aggregation events. Klucken et al. (2004) reported the efficacy of HSPs for the reduction of aggregated α-syn *in vitro* and *in vivo* studies. An interesting point of investigation is how aggregation-prone polypeptides escape protein quality control systems on the way to forming pathological aggregates. Indeed, HSPs may get trapped within aggregates as the rate of the aggregate formation increases with the progression of pathology, thus reducing the availability of these molecular chaperones. The kinetics of aggregation influence the ability of small HSPs to inhibit α-syn aggregation, and may indicate how these aggregates potentially evade HSP chaperone-action, as the aggregated protein-loads increase the burden on this system (Cox et al., 2016). Therefore, further investigation is needed into mechanisms of regulating HSP expression levels to offer neuroprotection *via* chaperone induction/co-induction.

Another approach to reducing the risk of α-syn aggregation inside cells utilizes an oligomer modulator called Anle138b [3-(1,3-benzodioxol-5-yl)-5-(3-bromophenyl)-1H-pyrazole] that inhibits the formation and accumulation of α-syn oligomers but does not interfere with the level of protein expression (Wagner et al., 2013). Importantly, the compound slowed disease progression in human A30P α-syn transgenic mice even when treatment started after the onset of disease-related symptoms (Levin et al., 2014). Indeed, both in mouse models of prion disease and in three different PD mouse models, Anle138b strongly inhibited oligomer accumulation, neuronal degeneration, and disease progression (Wagner et al., 2013). Interestingly, binding studies revealed that Anle138b does not bind the monomeric form of α-syn, and thus does not interfere with non-aggregated forms of the protein in the physiologic state. Additionally, no detectable toxicity was reported at therapeutic doses and the compound exhibited excellent oral bioavailability and blood-brain barrier (BBB) penetrance. A recent study by Deeg et al. (2015) reported that Anle138b and other diphenyl-pyrazole compounds exhibited significantly increased fluorescence upon binding to fibrillar α-syn structures, rendering this compound a promising fluorescent biomarker for investigation of aggregation dependent epitopes.

Early *in vitro* studies revealed numerous small molecule inhibitors of α-syn assembly. Masuda et al. (2006) tested 79 compounds belonging to 12 different chemical classes and found that compounds from seven of these classes (polyphenols, phenothiazines, polyene macrolides, porphyrins, rifamycins, Congo red and its derivatives, and terpenoids) inhibited α-syn filament assembly. Several molecules including, baicalein, delphinidin, dopamine chloride, and gallocatechin, were of particular interest, as they exhibited strong inhibitory properties against α-syn filament formation. Soluble α-syn oligomers formed instead in the presence of inhibitory compounds, probably through binding interactions with the C-terminal, suggesting this may be the mechanism through which filament formation is inhibited. These findings were corroborated by the evidence presented 2 years earlier by Zhu et al. (2004) who showed that baicalein both inhibited the formation of α-syn fibrils and disaggregated pre-existing α-syn filaments.

Several other small molecules have been reported to inhibit α-syn aggregation. Methylthioninium is one such example of an effective aggregation inhibitor of a-syn fibrillar inclusions both *in vitro* and *in vivo* that also exhibits the potential of rescuing behavioral deficits and ameliorating pathology in transgenic mouse models (Schwab et al., 2018). NPT100-18A interacts with the C-terminal domain of α-syn, displaces it from the membrane, reducing the formation of α-syn oligomers, and subsequently reduced neuronal accumulation of α-syn and decreased markers of cell toxicity. Treatment with NPT100-18A improved motor deficits in mThy1 WT α-syn transgenic mice in a dose-dependent manner (Wrasidlo et al., 2016). NPT200-11 is another inhibitor of α-syn aggregation comparable to NPT100-18A, which stabilizes α-syn conformers and blocks pathological misfolding of the protein. A small randomized, double-blind, single ascending dose phase 1 clinical trial evaluating the safety, tolerability, and pharmacokinetics of NPT200-11 was recently completed (Clinicaltrials.gov Identifier: NCT02606682). A fourth molecule is the human IgG1 fusion protein NPT088 that contains the General Amyloid Interaction Motif (GAIM) and is designed to simultaneously target multiple misfolded proteins. NPT088 treated Thy1-Hα-syn mice showed increased tyrosine hydroxylase levels concomitant with a significant reduction in proteinase K-resistant α-syn (Fisher et al., 2015). A multidose phase 1 clinical trial is currently underway to evaluate the safety and tolerability of NPT088 in patients with probable Alzheimer’s disease (NCT03008161).

Molecular tweezers have also been shown to be strong dissociative and anti-aggregation agents (Sinha et al., 2011, 2012). These molecules are relatively small molecules that inhibit protein interactions; CLR01 has shown great promise *in vitro* inhibiting α-syn aggregation across multiple studies (Sinha et al., 2012; Acharya et al., 2014; Schrader et al., 2016). CLR01 has shown to be effective at reducing α-syn-induced cell death *in vitro* and has also been shown to reduce α-syn proteasomal inhibition in zebrafish (Prabhudesai et al., 2012). This prompted the investigation of the safety of this lysine-specific tweezer in a mammalian system; not only was CLR01 found to be safe and well-tolerated in mice, but it was also shown to cross the BBB, ensuring delivery to the target tissue in subsequent studies (Attar et al., 2014). In a pre-clinical PD mouse model CLR01 was able to reduce motor symptoms and α-syn-related pathology (Richter et al., 2017), indicating it is an interesting candidate for the treatment of PD. Strengthening this evidence with more pre-clinical research would highlight the case for CLR01 and other molecular tweezers as candidates for PD clinical trials.

Immunotherapy is another promising strategy for the reduction of α-syn aggregates that is currently in clinical trials. Several studies have reported that both active and passive immunization against α-syn was neuroprotective in transgenic mouse models of PD (Masliah et al., 2005, 2011; Bae et al., 2013; Sanchez-Guajardo et al., 2013). These findings prompted the first phase 1 clinical trial with PRX002, a humanized IgG1 monoclonal antibody directed against epitopes near the C-terminus of aggregated forms of α-syn. Results from the phase 1a clinical trial reported a 96.5% reduction in the concentration of free serum α-syn (Schenk et al., 2017). A multiple-ascending dose study was performed in a phase 1b clinical trial to evaluate the safety and tolerability of PRX002 in idiopathic PD patients (Jankovic et al., 2018). The results of this study demonstrated significant target engagement evidenced by the reduction in free serum α-syn levels by up to 97% after a single infusion cerebrospinal fluid (CSF) penetration indicated by a dose-dependent increase in CSF PRX002 concentrations. All doses of PRX002 were found to be safe and tolerable, supporting the design of an ongoing phase 2 clinical trial (NCT03100149; Jankovic et al., 2018).

In contrast to PRX002, BIIB054 (Biogen) is a fully human-derived monoclonal antibody directed against the N-terminal epitope of α-syn that exhibits a high level of specificity for aggregated and fibrillar forms of α-syn with a more than 800-fold binding affinity for aggregated α-syn compared with monomeric forms of the protein (Weihofen et al., 2019). BIIB054 treatment was reported to reduce the spread of truncated α-syn variants to the contralateral cortex and rescued motor impairments by about 50% in α-syn PFF-inoculated WT mice (Weihofen et al., 2019). A recently-concluded phase 1 clinical trial of a single-ascending dose study of BIIB054 in PD patients and healthy volunteers, reported it was well tolerated at single doses up to 90 mg/kg, had a serum half-life of 28 days, and CSF concentrations achieved in healthy volunteers were 0.2% of those seen in plasma (Brys et al., 2019). Single doses of BIIB054 up to 45 mg/kg were well tolerated in PD patients, and the pharmacokinetic profile was comparable to that seen in healthy volunteers. BIIB054 is currently being evaluated in recently diagnosed PD patients in a multinational phase 2 clinical trial (NCT03318523).

Active immunization approaches have also been previously studied. AFFiRiS, developed AFFITOPE, a vaccine that consists of short synthetic α-syn peptide fragments. In the recently completed parallel-group phase 1 clinical trial, the AFF008 study series assessed the tolerability and safety of repeated subcutaneous administration of a synthetic α-syn mimicking epitope called AFFITOPE PD01A in a group of 24 patients randomized to receive either AFFITOPE PD01A low dose or high dose (NCT01568099). Similar results were observed in another phase 1 clinical trial study that used a different synthetic α-syn mimicking epitope called AFFITOPE PD03A (NCT02267434). At the screening, the average duration of PD after the initial diagnosis was between 1.6–2.3 years. The study design was comparable to that used with AFFITOPE PD01A, with 36 patients randomized to a low dose, high dose, or placebo group. Treatment was found to be safe and well-tolerated at both doses in PD patients. AFFITOPE PD01A elicited a clear dose-dependent immune response against the peptide itself and cross-reactivity against the α-syn targeted epitope over time. Overall results also reported a significant increase in antibody titer against PD01A. However, there was no significant immunogenicity of AFFITOPE PD03A when compared with controls.

Although antibodies are invaluable tools for synucleinopathy research, their high molecular weight undermines the viability of their therapeutic potential. This is especially important in neurological disorders as the BBB is a formidable obstacle to the systemic treatment of CNS diseases, because it impairs the passage of the vast majority of molecules, including antibodies, to the brain. Therefore, an interesting new avenue of research is that of gene-engineered antibodies called intrabodies and nanobodies, by expressing regions for antibody specificity separate from the full-length antibody. While a major concern about the use of immunotherapy in PD treatment is whether antibodies will have significant brain penetration to achieve sufficient target engagement, these engineered fragments might circumvent that issue, as they have higher brain penetrance and faster clearance. Additionally, because they can be synthesized in large quantities by bacterial or yeast systems, their production can be made more efficient and economical, providing further support for their viability. Zhou et al. (2004) reported scFv fragments, comprised of heavy variable and light variable domains linked by a short, flexible polypeptide sequence, that bind and stabilize monomeric forms of α-syn, thereby inhibiting the formation of insoluble high-molecular-weight α-syn species. Moreover, Zha et al. (2016) demonstrated that use of scFv W20 against common epitope of various toxic oligomeric species, reduced α-syn and mutant huntingtin protein aggregate load in PD and HD transgenic mice, and simultaneously reduced synaptic degeneration, neuroinflammation, and oxidative stress and significantly improved motor and cognitive deficits. Additionally, single domain antibodies called nanobodies might be an alternative to scFvs (Hamers-Casterman et al., 1993). Nanobodies have a higher solubility and a lower molecular weight compared with scFvs, rendering greater brain penetrance due to increased passage through the BBB. Their ability to recognize unique epitopes with subnanomolar affinity and their high production yield at a relatively low cost make them a useful class of biomolecules for research and therapeutic applications. A study on the nanobody NbSyn87 that has target specificity for residues 118–131 in the C-terminus of α-syn reported that it binds both monomeric and fibrillar forms of the protein, indicating that the epitope is accessible in the fibrillar state (Guilliams et al., 2013). The length of time of fibrillization influenced the apparent affinities of NbSyn87 for their epitopes. This finding suggests that the epitope on the α-syn C-terminus undergoes conformational changes during fibrillization and that nanobodies are able to target different, potentially pathogenic forms of aggregated α-syn.

Although neuro-immunotherapy presents as an elegant tool to inhibit the pathogenic spread of extracellular aggregated α-syn, the potential associated risks in both active and passive immunization, including systemic side reactions, need to be clearly elucidated. Neurodegenerative disease treatment requires further investigation into the multiple routes of vector administration, including direct injection, injection into the CSF, and intramuscular and intravascular administration. Subsequent studies will need to clearly evaluate the safety and risk associated with more invasive measures that efficiently transduce or treat smaller areas, compared with broader, less-invasive means of distribution associated with limited brain penetrance due to the need to bypass the BBB. Optimization of these technological challenges, including means to either mechanically or biologically bypass the BBB, achieve sufficient antibody levels in CNS to enable adequate target engagement, and direct elimination only against aggregated α-syn species, are needed to further evaluate important implications for future therapeutics.

### Enhancing Degradation of Intracellular α-Syn Aggregates

Autophagy is suggested to serve a significant role in the intracellular degradation of α-syn aggregates (Decressac et al., 2013). Once the autophagosome has formed around its target, a lysosome can merge with it, delivering its degradative enzymes to the enclosed, pathogenic cargo. Autophagosome-lysosome fusion events are mediated by Rab GTPases that recruit membrane-tethering complexes to reduce the spatial separation between the two compartments and SNARE proteins that drive the physically fusion of the bilayers (Nakamura and Yoshimori, 2017). Dysfunction of the ALP has been shown to correlate with increased accumulation of intracellular α-syn aggregates and induce the activation of nonclassical secretory pathways (Poehler et al., 2014). While the presence of intracellular α-syn aggregates may increase the pathologic burden on host neurons, neuron-released α-syn aggregates may enhance the cell-to-cell propagation of α-syn pathology and progression of disease (Lopes da Fonseca et al., 2015). Thus, enhancing autophagic processes may promote increased clearance of pathological α-syn and alleviate the intracellular burden on host neurons.

The mammalian target of rapamycin (mTOR), a component of protein complexes mTORC1 and mTORC2 are essential for cellular development and tissue regeneration, as well as the regulation of apoptosis and autophagy (Maiese et al., 2013). Rapamycin and its analogs work *via* mTOR inhibition to induce autophagy. This class of drugs has been shown to have neuroprotective effects primarily due to its ability to promote increased clearance of α-syn through the induction of autophagic processes (Webb et al., 2003; Crews et al., 2010; Bové et al., 2011; Decressac et al., 2013; Maiese et al., 2013). However, rapamycin has limited utility because it lacks specificity, and acts on other essential pathways involved in immunosuppression, and thus is not a viable drug candidate for PD where long-term treatment would be necessary. Due to rapamycin’s therapeutic limitations, researchers have investigated other compounds that promote autophagy. For example, trehalose is a sugar molecule present in many organisms that acts through an mTOR-independent pathway, and has been shown to enhance autophagy through increased lysosomal biogenesis, leading to the corresponding increased clearance of protein aggregates (Sarkar et al., 2007). Another strategy to achieve inhibition of mTOR uses a modulator of the mitochondrial pyruvate carrier (MPC) called MSDC-0160 to reduce pyruvate transport into the mitochondria. MSDC-0160 causes alterations in mitochondrial metabolism induced by MPC inhibition that result in mTOR inhibition and upregulation of autophagy in neurons (Ghosh et al., 2016). It has been shown to protect midbrain dopaminergic neurons from MPP+-induced cell death and nigral dopaminergic neurons in a “chronic” genetic mouse model of PD, and enhance autophagy in a model of α-syn-induced toxicity in *C. elegans* (Ghosh et al., 2016). These findings warrant further investigations to determine whether MPC inhibition may present promise as a useful avenue for future therapeutics, and may provide insight into the development of other MPC modulators as potential disease-modifying agents for PD.

## Conclusion

Substantial evidence supports the identification of α-syn is a key player in the initiation and progression of neurodegeneration in PD pathogenesis. Several pre-clinical therapeutic modalities targeting pathological α-syn have revealed promising results. Current approaches include treatments designed to inhibit the synthesis, aggregation, or uptake of abnormal α-syn and enhance extracellular protein clearance mechanisms. α-syn immunotherapy and small molecule-based dissociation of aggregates have garnered significant interest as potential methods that might slow or halt the progression of the disease. However, further research is needed to optimize our understanding of the clinic-pathological relationship between the various species of α-syn and the development of PD. While caution is warranted when manipulating global α-syn levels, we conclude that targeting toxic α-syn seems a compelling strategy for therapeutic targets in PD.

## Author Contributions

CF and NB-V wrote the initial draft. NB-V and RW-M revised and produced the final version of the manuscript.

## Conflict of Interest

The authors declare that the research was conducted in the absence of any commercial or financial relationships that could be construed as a potential conflict of interest.
